# The roles of aromatic residues in the glycine receptor transmembrane domain

**DOI:** 10.1186/s12868-018-0454-8

**Published:** 2018-09-06

**Authors:** Bijun Tang, Sarah C. R. Lummis

**Affiliations:** Department of Biochemistry, University of Cambridge, Cambridge, UK

**Keywords:** Ligand-gated ion channel, Cys-loop receptor, Aromatic amino acid, Mutagenesis, Electrophysiology

## Abstract

**Background:**

Cys-loop receptors play important roles in fast neuronal signal transmission. Functional receptors are pentamers, with each subunit having an extracellular, transmembrane (TM) and intracellular domain. Each TM domain contains 4 α-helices (M1–M4) joined by loops of varying lengths. Many of the amino acid residues that constitute these α-helices are hydrophobic, and there has been particular interest in aromatic residues, especially those in M4, which have the potential to contribute to the assembly and function of the receptor via a range of interactions with nearby residues.

**Results:**

Here we show that many aromatic residues in the M1, M3 and M4 α-helices of the glycine receptor are involved in the function of the receptor. The residues were explored by creating a range of mutant receptors, characterising them using two electrode voltage clamp in *Xenopus* oocytes, and interpreting changes in receptor parameters using currently available structural information on the open and closed states of the receptor. For 7 residues function was ablated with an Ala substitution: 3 Tyr residues at the extracellular end of M1, 2 Trp residues located towards the centers of M1 and M3, and a Phe and a Tyr residue in M4. For many of these an alternative aromatic residue restored wild-type-like function indicating the importance of the π ring. EC_50_s were increased with Ala substitution of 8 other aromatic residues, with those in M1 and M4 also having reduced currents, indicating a role in receptor assembly. The structure shows many potential interactions with nearby residues, especially between those that form the M1/M3/M4 interface, and we identify those that are supported by the functional data.

**Conclusion:**

The data reveal the importance and interactions of aromatic residues in the GlyR M1, M3 and M4 α-helices, many of which are essential for receptor function.

**Electronic supplementary material:**

The online version of this article (10.1186/s12868-018-0454-8) contains supplementary material, which is available to authorized users.

## Background

Cys-loop receptors, which include nicotinic acetylcholine (nACh), 5-HT_3_, GABA_A_, and glycine (Gly) receptors, are pentameric ligand-gated ion channels (pLGIC) responsible for fast excitatory and inhibitory synaptic neurotransmission in the central and peripheral nervous systems [1–4]. Members of this family are pentameric, with each of the subunits having an extracellular domain (ECD), a transmembrane domain (TMD), and an intracellular domain. Molecules that activate these receptors bind at the interface between two adjacent subunits in the ECD, triggering a conformational change that ultimately opens the ion channel in the TMD.

Recent high resolution structural data from both eukaryotic and prokaryotic pLGICs have begun to clarify their mechanism of action [5–12], but, as these are only snapshots in a particular state, much still remains to be discovered. The structures do, however, allow us greater insight into the specific roles of amino acids in these important proteins, which should ultimately allow us to understand how they contribute to protein function. Gly receptors (GlyR), especially α1 GlyR, have been particularly well examined to date. They are unusual in that they can function as homomeric proteins, thus allowing easier interpretation of functional data following mutagenesis. They also express well in heterologous systems, and indeed have proved to be the vertebrate pLGIC of choice for structural studies: there are more high resolution GlyR structures available than for any other Cys-loop receptor. Thus these proteins are good candidates for this type of study.

**Fig. 1:**
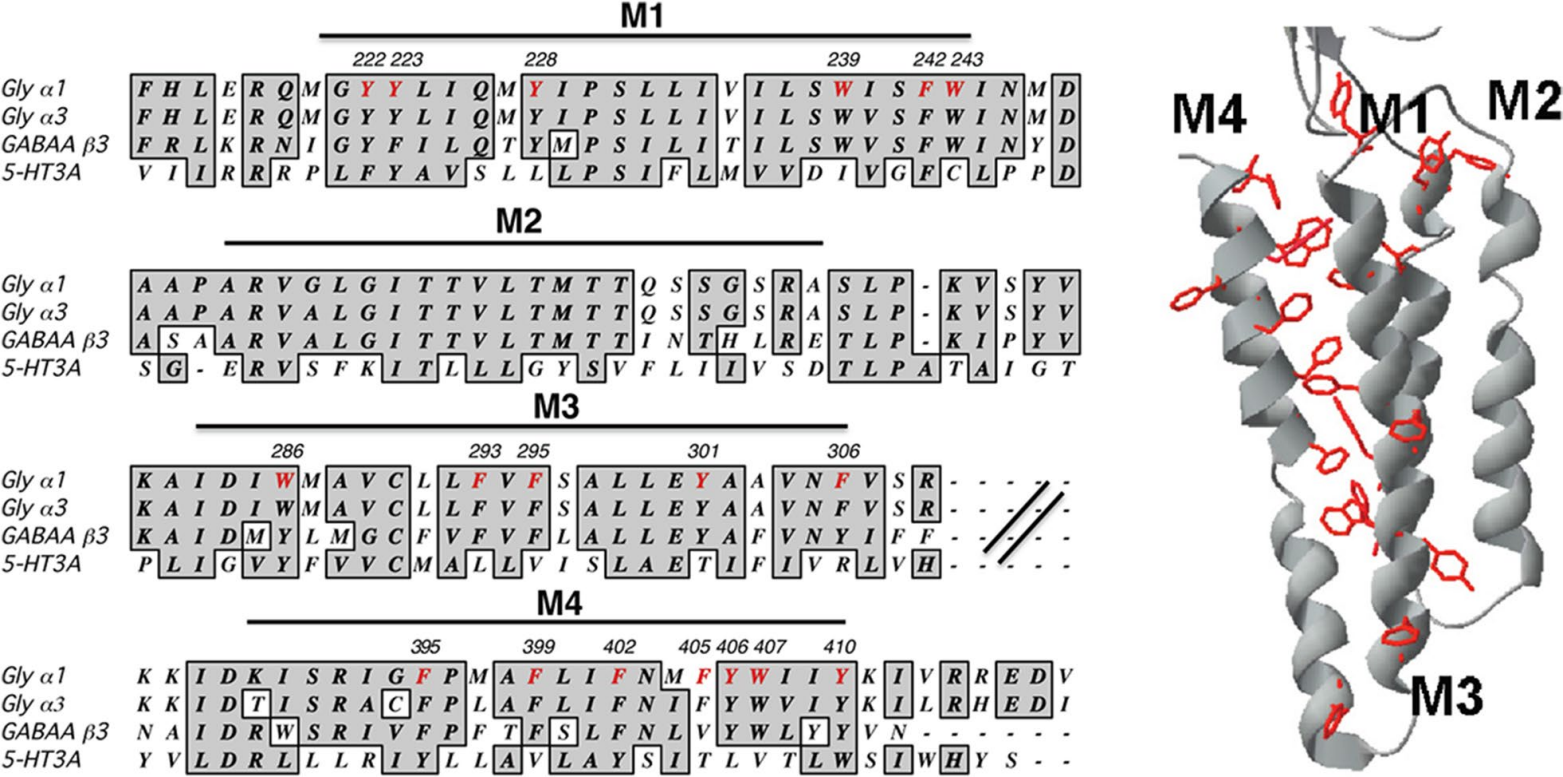
Alignment of the transmembrane domain of the GlyR α1 subunit with those from a range of other pLGIC subunits showing the aromatic residues examined in this study (in red), and the location of residues in the TMD of one of the 5 subunits that constitute the receptor, showing that many are located at the M1/M3/M4 interface

It has long been appreciated that aromatic residues are important in Cys-loop receptors, and in the GlyR many studies have demonstrated that the aromatic residues in the ECD play roles in the function, assembly, stability and expression of receptors [13–18]. Of particular importance are those aromatics clustered in the agonist or orthosteric binding site, which constitute the “aromatic box” found in all pLGIC. There are also many aromatic residues in the TMD (Fig. 1) and there have been a range of studies on these, with most of them being concerned with the residues in M4. Studies suggest that M4 interactions with M1 and M3 in some pLGICs are necessary to promote effective interactions between the M4 C-terminus and the Cys-loop, which are located at the interface of the ECD and the TMD, and are important in coupling agonist binding to channel opening [19, 20]. It is also possible that there are more direct links, for example W418 in the nAChR M4 α-helix has recently been shown to interact directly with S226 on the adjacent M1 α-helix, stabilizing the open state [21]. Thus understanding the interactions made by aromatic residues is an important step in understanding the mechanism of activation of pLGICs. Here we extend previous studies on the GlyR TMD aromatic residues, which were performed before the structure of this protein was available, and interpret the data using structural information now available from open and closed states of the GlyR. The data show that many of these residues play important roles in the function of the receptor.

## Methods

### Oocyte maintenance

*Xenopus* oocytes were purchased from EcoCyte Bioscience (Dortmund, Germany) and stored in ND96 (96 mM NaCl, 2 mM KCl, 1.8 mM CaCl_2_, 1 mM MgCl_2_, 5 mM HEPES, pH 7.5) containing 2.5 mM sodium pyruvate, 50 mM gentamycin and 0.7 mM theophylline.

### HEK293 cell culture

Human embryonic kidney (HEK) 293 cells (ECACC 85120602 Sigma Aldrich, UK) were maintained on 90 mm tissue culture plates at 37 °C and 7% CO_2_ in a humidified atmosphere. They were cultured in DMEM:F12 with GlutaMAX™ I media (Dulbecco’s Modified Eagle’s Medium/Nutrient Mix F12 (1:1), Invitrogen, Paisley, UK) containing 10% foetal calf serum. For immunofluorescent studies cells on cover slips were transfected using polyethyleneimine (PEI). 30 µl PEI (1 mg/ml), 5 µl cDNA and 1 ml DMEM were incubated for 10 min at room temperature, added drop wise to an 80–90% confluent plate, and incubated for 2–3 days before use.

### Receptor expression

cDNA was cloned into pGEMHE for oocyte expression, and pcDNA3.1 (Invitrogen, Paisley, UK) for expression in HEK 293 cells. Mutagenesis was performed using QuikChange (Agilent Technologies Inc., CA, USA); primers are shown in Additional file 1: Table S1. cRNA was in vitro transcribed from linearised pGEMHE cDNA template using the mMessage mMachine T7 Transcription kit (Ambion, Austin, TX, USA). Oocytes were injected with 50 nl of ~ 400 ng µl^−1^ cRNA, and currents were recorded 1–4 days post-injection.

### Electrophysiology

This was similar to our previous work [14, 15] although here we used a Robocyte voltage-clamp system (Multichannel systems, Germany). Briefly *Xenopus* oocytes were clamped at − 60 mV, and oocytes perfused with saline at a constant rate of 1 ml min^−1^. Drug application was via a simple gravity fed system calibrated to run at the same rate. Extracellular saline contained (mM), 96 NaCl, 2 KCl, 1.8 CaCl_2_, 1 MgCl_2_ and 5 HEPES; pH 7.4 with NaOH). Analysis and curve fitting was performed using Prism v4.03 (GraphPad Software, San Diego, CA, USA, www.graphpad.com). Concentration–response data for each oocyte were normalised to the maximum current for that oocyte. Statistical significance was determined using an ANOVA with a Dunnetts multiple comparison post test.

### Immunocytochemistry

This was as described previously [22]. Briefly, transiently transfected fixed cells were incubated in anti-glycine α1 receptor antisera (C-15; Santa Cruz Biotechnology). Biotinylated anti-rabbit IgG (Vector Laboratories, CA, USA) and fluorescein isothiocyanate avidin D (Vector Laboratories) were then used to detect bound antibody. Immunofluorescence was observed using a Leica fluorescent microscope.

### Structures

PDBs 5VDH (open GlyR), 5CFB (closed GlyR), 4COF (GABA_A_R) and 4HIF (Gloeobacter ligand-gated ion channel, GLIC) were downloaded from the PDB database and viewed and/or mutated using PyMol or Swiss-PDBViewer. The GlyR structures are those of the homomeric α3 GlyR, but the majority of residues, and in particular all the aromatic residues studied here, are identical to those in the α1 GlyR (Fig. 1).

## Results

**Fig. 2:**
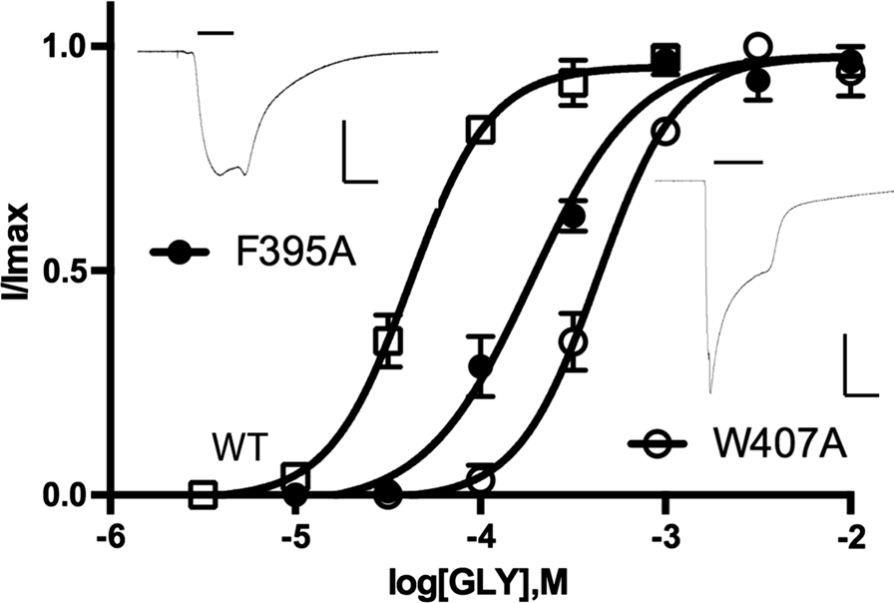
Example concentration response curves and maximal current traces for WT and two mutant GlyR. Data = mean ± SEM, n = 4–8. Parameters obtained from these curves are shown in Table 1. Scale bars are 2 μA and 10 s

**Fig. 3:**
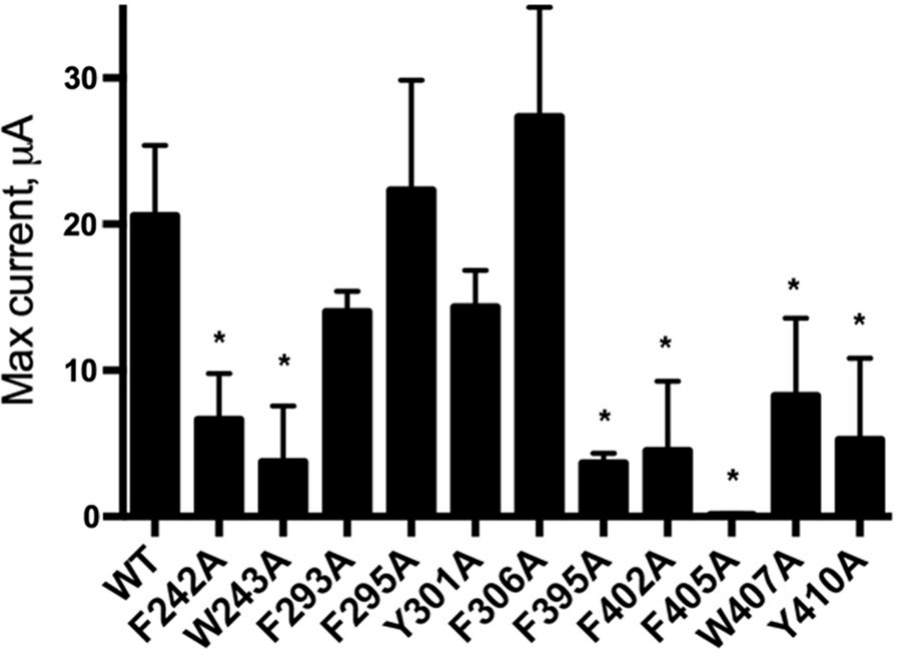
Maximal currents elicited by glycine are lower in mutants with Ala substitutions to aromatics in M1 and M4. Data = mean ± SEM, n = 4–8, * = significantly different to WT, p < 0.05, ANOVA with Dunnetts post-comparison test

To probe the roles of the aromatic residues in the transmembrane α-helices of the GlyR, we mutated each to Ala, and determined changes in functional characteristics following expression in *Xenopus* oocytes. We also mutated some of the more sensitive residues to an alternative aromatic residue, to clarify possible interactions with the π ring. Example concentration response curves and current traces are shown in Fig. 2. Mutation of seven of the nineteen aromatic residues to Ala resulted in nonfunctional receptors (Table 1), and, apart from one (F295A), all others resulted in increased EC_50_s and/or lower maximal currents (Fig. 3), indicating these residues are important for GlyR expression and/or function. The data from each transmembrane α-helix are discussed below.

**Table 1:** EC_50_s of WT and mutant GlyR

Mutant	pEC_50_ (M)	EC_50_ (μM)	Mutant	pEC_50_ (M)	EC_50_ (μM)
WT	4.380 ± 0.04	42	F293A	3.559 ± 0.06*	276
			F293Y	3.901 ± 0.02*	125
*M1*			F295A	4.339 ± 0.07	46
Y222A	NR		Y301A	3.463 ± 0.09*	345
Y222F	3.519 ± 0.03*	303	F306A	3.795 ± 0.03*	160
Y223A	NR				
Y223F	3.721 ± 0.07*	190	*M4*		
Y228A	NR		F395A	3.724 ± 0.05*	189
Y228F	4.466 ± 0.07	34	F399A	NR	
W239A	NR		F399Y	3.846 ± 0.04*	143
W239F	4.248 ± 0.04	57	F402A	4.183 ± 0.03	66
F242A	3.803 ± 0.07*	157	F402Y	3.725 ± 0.01*	188
W243A	3.764 ± 0.06*	172	F405A	2.961 ± 0.07*	1095
			F405Y	3.718 ± 0.07*	192
*M3*			Y406A	NR	
W286A	NR		W407A	3.366 ± 0.04*	430
W286Y	2.351 ± 0.05*	445	Y410A	3.489 ± 0.16*	324

Data = mean ± SEM, n = 3–8; * = significantly different to WT, p < 0.05 ANOVA with Dunnetts multiple comparison post-test; NR = non-responsive

### Aromatic residues in M1

**Fig. 4:**
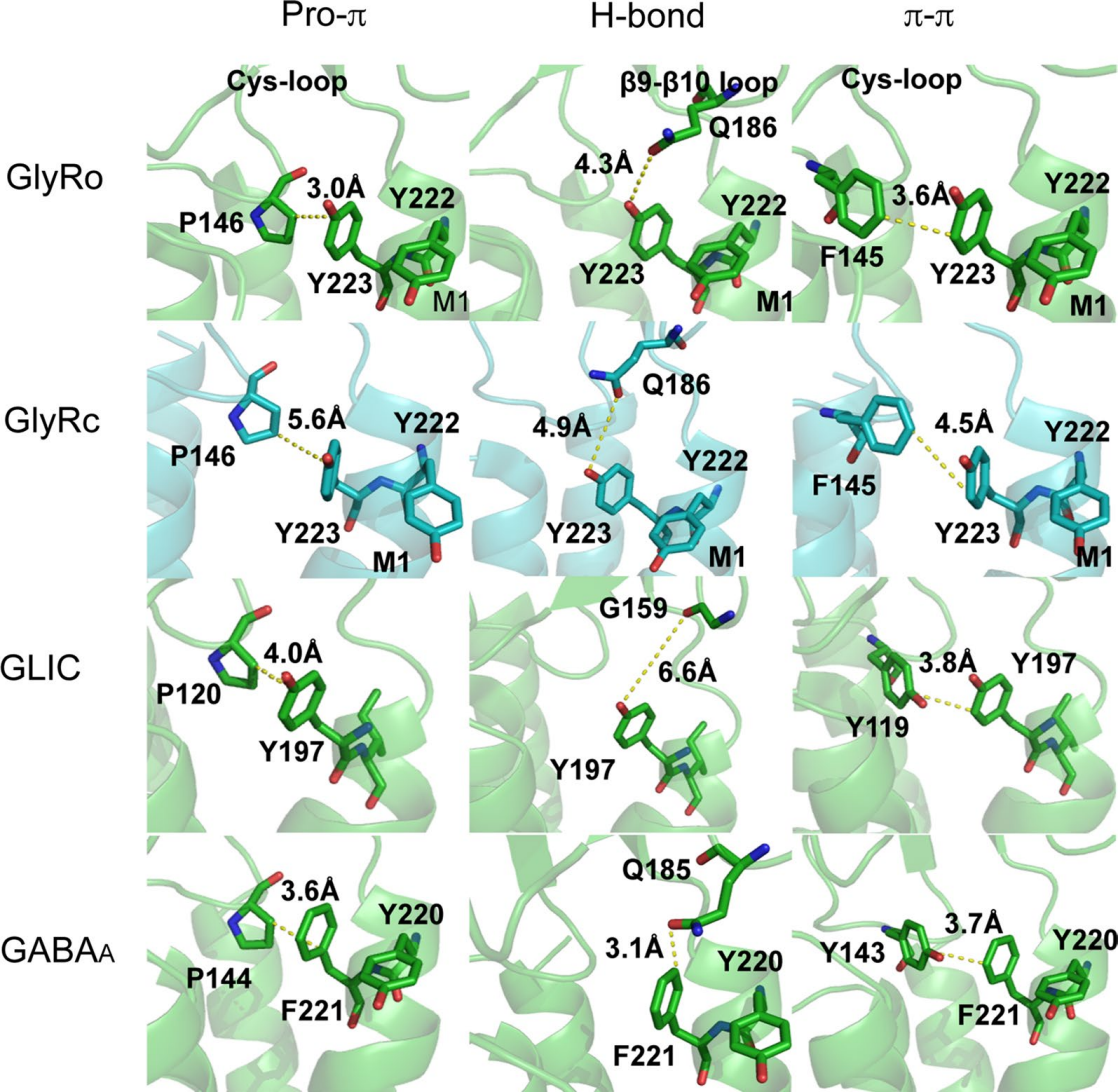
Structures of Gly, GLIC and GABA_A_ receptors showing possible interactions of Y222 and Y223 or equivalent residues. GlyRo = GlyR in open state; GlyRc = GlyR in closed state

The aromatic residues at the top of the M1 α-helix are more sensitive than those towards the intracellular side: Y222 and Y223 at the extracellular side result in non-functional receptors when substituted with Ala, despite being expressed (Additional file 2: Figure S1). When these Tyrs are replaced with an alternative aromatic residue, function is retained, but with increased EC_50_s (Table 1). Structural data show Y223 could interact with many neighbouring residues, although Y222 probably only with Y223 (Fig. 4).

**Fig. 5:**
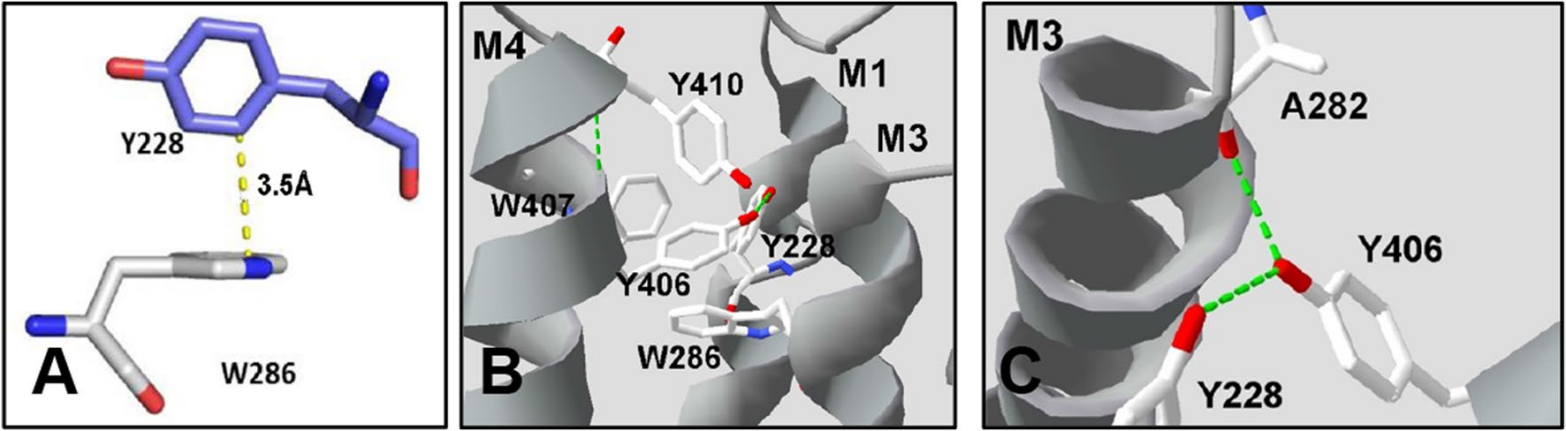
**A** Y228 and W286 are well positioned to form a T-type π–π interaction; **B** These residues, with Y406,W407 and Y410, form an aromatic cluster at the top of the M1/M3/M4 interface. **C** Y406 could form a hydrogen bond (green dashes) with either A282 or Y228; the data better support the former

**Fig. 6:**
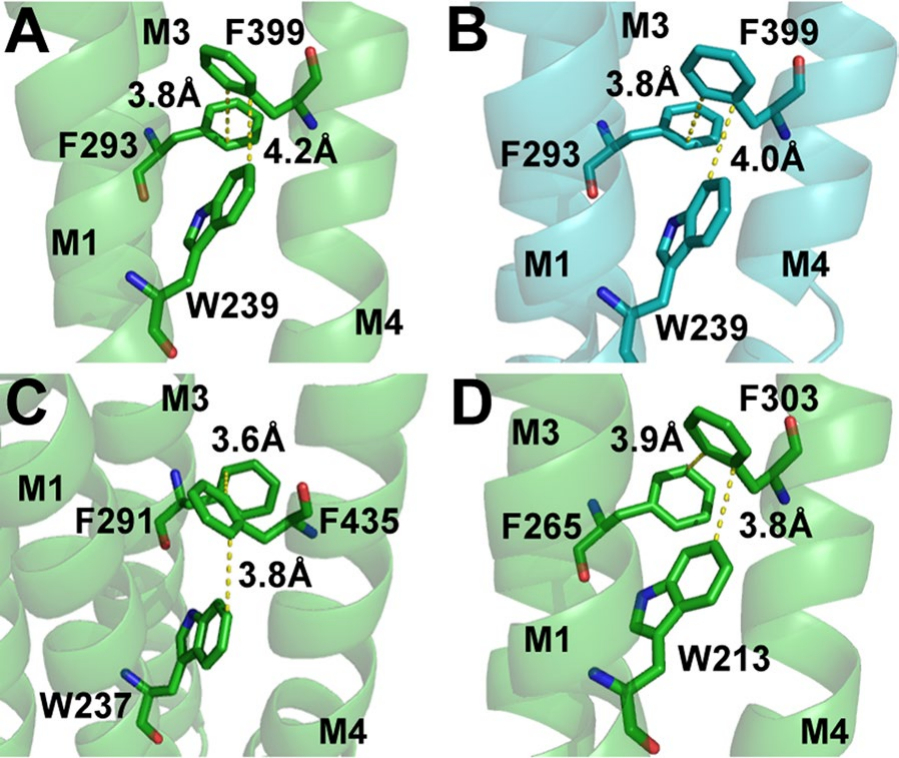
GlyR in the open (**A**) and closed (**B**) states show similar interactions between W239, F293 and F399. Structural data show equivalent residue interactions are possible in the GABA_A_ receptor (**C**) and GLIC (**D**)

**Fig. 7:**
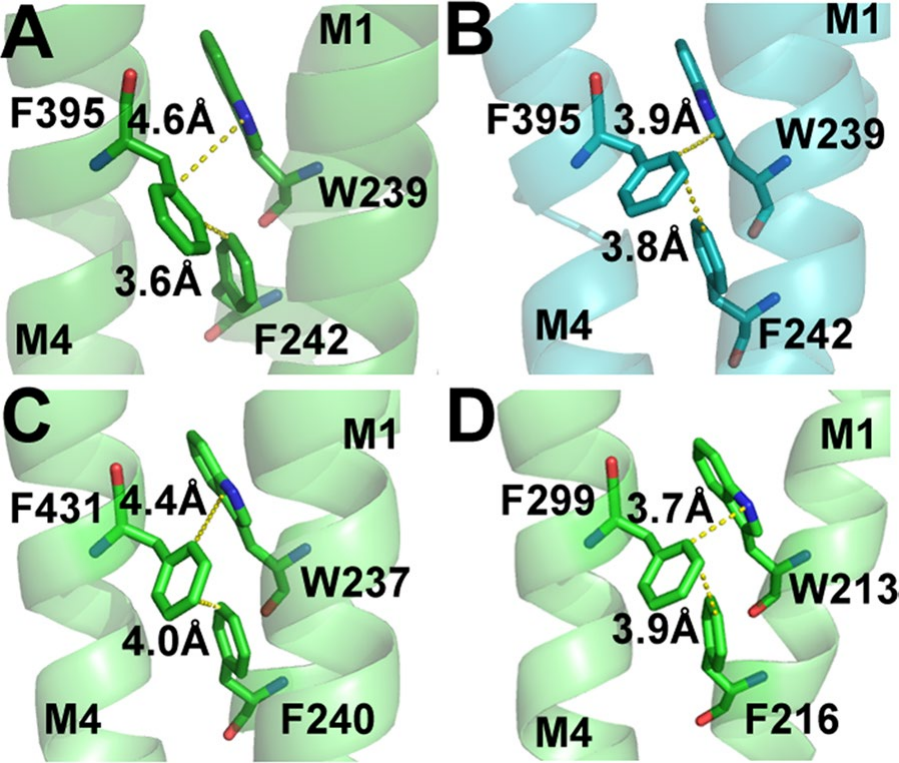
Distances between W239 and F395 differ in the open (**A**) and closed (**B**) states, although those between F395 and F242 do not. Structural data show equivalent residue interactions in this region are possible in the GABA_A_ receptor (**C**) and GLIC (**D**)

Y228 and W239, which are towards the centre of M1, also result on non-functional receptors when substituted with Ala, but here an alternative aromatic results in EC_50_ values similar to wild type receptors. A number of interactions with neighbouring residues are revealed from the structural data (Figs. 5, 6, 7).

**Fig. 8:**
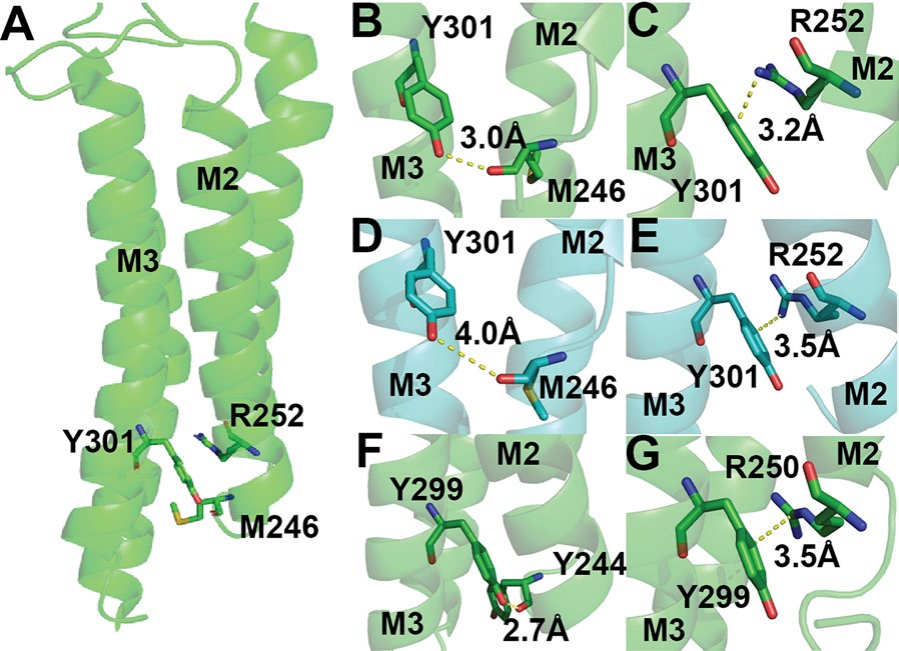
Y301 in M3 (**A**) could form a hydrogen bond with M246 and/or a cation–π interaction with R252 in the open state (**B**, **C**) but probably not the former in the closed state (**D**, **E**). Similar interactions are possible in the GABA_A_ receptor (**F**, **G**)

**Fig. 9:**
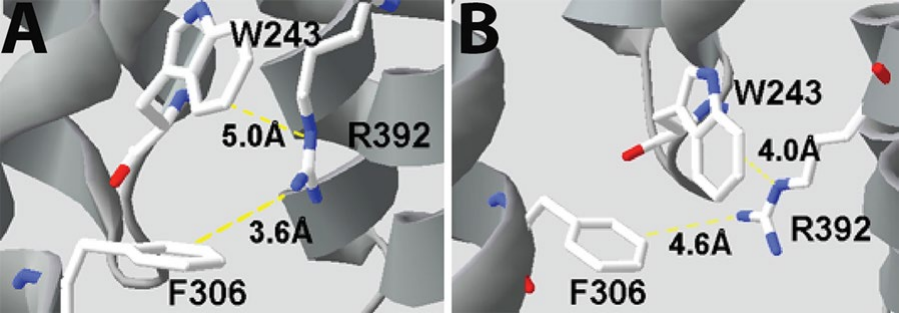
F306 could form a cation–π interaction with R392 in the open state (**A**), but R392 is more likely to do this with W243 in the closed state (**B**)

F242 and W243 differ from the other M1 aromatics as they do result in functional receptors when substituted with Ala, but both have increased EC_50_s values (Table 1), and decreased maximal responses (Fig. 3), indicating that a residue with a π ring is not essential at these locations, although it is preferred. Possible interactions with F395 and R392 respectively are revealed from the structural data (Figs. 7, 8, 9).

### Aromatic residues in M3

Only one aromatic residue in M3 (W286) resulted in non-functional receptors when substituted with Ala, although substitution with an alternative aromatic here yielded receptors with a large (~ 10 fold) increase in EC_50_ indicating the importance of this Trp. Ala substitution of 3 of the 4 other aromatic residues in this loop resulted in increases in EC_50_ (3–8 fold), while F295A- containing receptors were wild type-like (Table 1). Thus again these data suggest that a residue with a π ring is favored (apart from at position 295), but not essential. All of these receptors had maximal currents that were similar to wild type (Fig. 3). Various potential interactions of these M3 aromatics were observed from the structural data (Figs. 5, 6, 7, 8, 9).

### Aromatic residues in M4

Ala substitution of the aromatic residues in M4 have been previously reported [17], and our data are similar, revealing non-functional receptors with F399A and F406A-containing receptors, and increases in EC_50_ for most of the others, showing the impact of the π ring. All functional receptors also had decreased maximal currents compared to wild-type receptors (Fig. 3). To further explore some of these we substituted an alternative aromatic, which for some partially restored function (Table 1). F402 was unusual here in that substitution with Ala resulted in wild-type-like receptors but with Tyr resulted in an increased EC_50_. Interaction of these residues with many of their neighbours was observed from the structural data (Figs. 5, 6, 7, 8).

## Discussion

In order to understand the roles of individual residues in receptors, it is critical to combine both structural and functional data. Here we have used such data to understand the interactions and importance of aromatic residues in the GlyR TMD. Aromatic residues are among many hydrophobic residues located, as would be expected, in the transmembrane α-helices, and many of these hydrophobic residues play some role in the assembly and/or correct function of the TMD. Aromatic residues, however, are especially important, as they have the potential for more interactions with adjacent residues because of their π rings. Indeed we observed that these residues frequently contribute to aromatic networks that exist between the different α-helices that constitute the TMD, and many are necessary for the efficient functioning of the protein; these interactions and networks are discussed in more detail below.

### Y222 and Y223

Y222 and Y223 are located at the N-terminus of the M1 α-helix of the GlyR. Our data indicate both these residues are important for receptor function, with Ala substitution ablating function, although this is retained, albeit with an increase in EC_50_, with a Phe substitution. Our data do differ from a previous study [17] who found that Ala substitution did not ablate function. We cannot currently explain this, although in that study currents were reduced (e.g. Y223A currents were 14% of WT) and no EC_50_ data were given, so it is difficult to directly compare them with the current work. We did, however, observe that both Y222A and Y223A-containing GlyR were expressed in HEK293 cells, suggesting aromatic residues at these locations are not essential for receptor expression.

The structural data indicate that the only interaction that Y222 could make with adjacent residues is a π–π interaction with Y223. Such an interaction could help stabilise Y223, which has the potential for multiple interactions as shown in Fig. 4. Firstly it could form a bond between the aromatic ring of Y223 and the polarized CH in P146 (a CH–π bond) in the Cys-loop. A Pro in the Cys-loop is essential for pLGIC function, and it has been proposed this family of proteins should be renamed Pro-loop receptors because of the conservation of this Pro in all pLGICs [23]. Previous studies suggest this Pro may form a critical cis peptide bond [24, 25]; the intrinsically higher cis bias of Pro peptide bonds compared to other residues is consistent with this proposal. It is possible that a CH-π bond here could assist in stabilizing a cis peptide bond, and/or this interaction could hold the Cys-loop in a position or orientation that allows gating: the distance between Y223 and P146 differs in the open and closed states, and thus an interaction here might be important to stabilize the GlyR in the open state. GLIC and GABA_A_R structures show similar distances between equivalent residues, indicating that similar interactions may occur in these and perhaps all pLGICs.

Y223 could also form a hydrogen bond with Q186, located on the β9-β10 loop, another important region for channel gating (Fig. 4). However, the distance between these residues suggest a hydrogen bond here would be weak, and it is not conserved in GLIC and GABA_A_R. In addition the EC_50_ difference when the OH in Tyr is removed (i.e. in Phe) is small (< 4 fold). Thus we conclude there is no hydrogen bond here.

Y223 is also an appropriate distance and orientation from F145 in the Cys-loop to form a π–π interaction (Fig. 4). F145 in the GlyR has been shown to be important in providing a hydrophobic framework for a strong electrostatic interaction between D148 in the Cys-loop and R218 in M1 [26], and an interaction with Y223 could assist in the correct positioning of F145. Conservation of similar residues between GlyR, GLIC and GABA_A_R provide some support for this hypothesis, although the distances observed suggest that this interaction is less likely than that with P146, although both could contribute to Cys-loop stability.

### Y228, W286, Y406, W407 and Y410

Y228 and W286 are located at the N-terminal end of the M1 α-helix and M3 α-helix respectively, and substitution with Ala ablates function, consistent with previous studies that show no surface expression [17], while substitution with an alternative aromatic (Phe or Tyr respectively) results in receptors that function well (Table 1). These data suggest that π rings are important in residues at these locations for receptor assembly, and the structure reveals Y228 and W286 are ideally placed to form a T-type π–π interaction (Fig. 5A). Replacement of Y228 with Phe would have little effect on such an interaction, consistent with a WT-like EC_50_ of Y228F mutant GlyR, while a W286 to Tyr substitution would have a greater impact, as the orientations of the 2 π rings would likely be less than ideal; our data showing an ~ 10 fold increase in EC_50_ in W286Y-containing GlyR are again consistent with this interpretation. These residues are not, however, the only aromatics in this location: Y406, W407 and Y410 at the C-terminal end of the M4 α-helix are close to Y228 and W286, and these five residues form an aromatic cluster between the M1, M3 and M4 α-helices at the extracellular side of the TMD (Fig. 5B). The loss of Gly-elicited responses in Y406A-containing GlyR, consistent with previous studies that show no surface expression [17], and increase in EC_50_ in W407A-and Y410A-containing GlyR, supports contributions of these aromatic residues to the assembly and function of the receptor as previously proposed [17]. These authors also demonstrated that these latter residues contribute to assembly as shown by their lower expression levels (and resulting lower maximal currents) with Ala substitution, and our data is similar, supporting this proposal (Fig. 3). They also suggested that Y228 interacts with F293, F402 and F405, but we now know that Y228 is too far from these residues for any interaction. Similarly Y406, W407 and Y410 were aberrantly suggested to face and interact with the lipid bilayer, stabilizing the M4 α-helix. The structure reveals they actually face the interface of M1, M3 and M4, and thus do likely have a role in stabilizing M4, but by interacting with M1 and M3 α-helices and not the lipid.

The structural data also predict a hydrogen bond between Y228 and Y406, but the WT-like response of Y228F containing GlyR indicates that if there is a hydrogen bond here it does not contribute to receptor function. Y406 could alternatively hydrogen bond with the backbone of A282 (Fig. 5C) and, given the increase in EC_50_ with Y406F containing GlyR, we propose this interaction does occur, and has a role in linking M1 and M4 that is perhaps important for assembly.

These suggested interactions are supported by studies in the GABA_A_R, where Y474 (equivalent to Y406) is predicted to hydrogen bond with Y289 (Y228), and Y474 has a likely π–π interaction with Y346 (W286) on M3 [27]. In GLIC, these aromatic residues are not conserved. However, I202 (Y228), I259 (W286), L310 (Y406) and A311 (W407) could perhaps form a hydrophobic patch that plays a similar role.

### W239, F293 and F399

These residues are located in the M1/M3/M4 interface just below the aromatic cluster described above. Mutation of W239 and F399 to Ala ablated function, consistent with previous studies that show no surface expression [17], while function was retained with an alternative aromatic (Phe or Tyr respectively; Table 1), indicating a residue with a π ring is necessary for assembly. An aromatic at F293 is less important: F293A containing GlyR had an ~ 4 fold increase in EC_50_ which was reduced to ~ 2 fold with a Tyr substitution, suggesting that a small aromatic residue is preferred but not essential. We propose these residues interact with each other via π–π interactions (Fig. 6), which plays a role in stabilizing the transmembrane domain of the glycine receptor. Haeger et al. [17] also suggested there are interactions here, but details were inaccurate due to the lack of structural data. Such interactions are further supported by GLIC and GABA_A_ receptor structural data: the Phe equivalent to F399 in M4 could interact with the Trp equivalent of W239 in M1 and the Phe equivalent of F293 in M3.

### W239, F242 and F395

These residues on M1 (W239 and F242) and M4 (F395) could form π–π interactions (Fig. 7A, B) with W239 linking this group of aromatic residues with the one described above. W239 is the most sensitive of these residues to Ala substitution, where function is ablated as described above, whereas there are EC_50_s increases of 3–4 fold and decreases in maximal currents (as previously reported [17]) for F242A and F395A-containing GlyR (Table 1, Fig. 3) indicating roles in both assembly and function. WT-like responses with W239F containing GlyR suggest an aromatic residue is important here, and the altered distance between this residue and F395 in the open and closed states of the receptor suggest a π–π interaction that plays a role in stabilizing the open state. Data from other pLGIC indicate the equivalent residues in GLIC F216(M1) and F299(M4), ELIC F222(M1) and F303(M4) and GABAρR F303 (M1) and F463 (M4) may have similar interactions (e.g. Fig. 7C, D).

### Y301, F306 and W243

These residues are located towards the intracellular side of the TMD. Y301 and F306 are on M3 and have not been previously studied. Our data indicate they play a role in function but not expression, as Ala substitutions resulted in changes to EC_50_s (Table 1) but not I_max_ (Fig. 3), while W243 on M1, where Ala substitution results in changes to both EC_50_ and I_max_ could have roles in both expression and function.

Ala substitution of Y301 results in an 8 fold increase in EC_50_. This residue has the potential to hydrogen bond with the backbone of M246 on M2 and/or form a cation–π interaction with R252, also on M2 (Fig. 8A–E). The distance between Y301 and M246 varies by 1Å between the open and closed states, whereas the distance between Y301 and R252 is similar. These data suggest a possible role of the hydrogen bond in stabilising the open state, while the cation–π interaction links M2 or M3, which could be important for information transfer between the transmembrane α-helices. Equivalent residues in the GABA_A_ receptor could also form both the hydrogen bond and the cation–π interaction (Fig. 8F, G), providing some support for our suggestion, although no equivalent aromatic residue is present in GLIC.

Ala substitution of F306 or W243 both result in an ~ 4 fold increase in EC_50_. F306 is sufficiently close to R392 (M4) to form a cation–π interaction in the open but not the closed state, while W243 could form a cation–π interaction in the closed but not the open state (Fig. 9); such interactions could play a role in receptor opening and/or closing. Phe and Trp residues are conserved at these locations in the GABA_A_ receptor and GLIC, supporting this hypothesis. W243 is also less than 4 Å from the backbone of W239, and thus could help stabilize this residue, which, as discussed above, is important for receptor expression.

### F295 and F405

Ala substitution of F295 and F405 produce receptors that are similar to WT, and the structural data reveal both these residues face away from the core of the protein into the lipid bilayer. This indicates, as expected, that any hydrophobic residue could likely be located at these positions, as the presence of a π ring is not necessary.

## Conclusions

In conclusion we have used functional data combined with structural information to reveal the importance and interactions of the aromatic residues in the TMD of the glycine receptor. Some of these residues, especially those in M4, have been previously studied, but without the structural data it was previously not possible to accurately determine how they might interact with adjacent residues. The new information we have provided allow the clarification of the roles of these residues, and will also contribute to a full understanding of the mechanism of action of these critical neuronal proteins.

## Additional files

**Additional file 1: Table S1.** Oligonucleotides used to create the Ala mutants of each transmembrane aromatic residue.

**Additional file 2: Figure S1.** Example immunofluorescent images from WT F222A, and F223A GlyR shows all were expressed in HEK293 cells.

## Abbreviations

nACh: nicotinic acetylcholine
pLGIC: pentameric ligand-gated ion channels
ECD: extracellular domain
TMD: transmembrane domain
GlyR: glycine receptor
DMEM: Dulbecco’s Modified Eagle’s Medium
HEK: human embryonic kidney
GLIC: gloeobacter ligand-gated ion channel
