# Supplementary material for: The roles of aromatic residues in the glycine receptor transmembrane domain
Source: BMC Neurosci. 2018 Sep 6;19:53. doi: 10.1186/s12868-018-0454-8 (PMC6127993; doi:10.1186/s12868-018-0454-8)
Supplement: Supplementary file 1 — Additional file 1: Table S1. Oligonucleotides used to create the Ala mutants of each transmembrane aromatic residue. [file 12868_2018_454_MOESM1_ESM.pdf]

Table S1: Oligonucleotides used to create the Ala mutants of each transmembrane aromatic residue. The Ala codons were replaced with the appropriate alternative codons where we also created non Ala substitutions

Y222A	Forward	CTAGAACGGCAGATGGGCGCCTACCTGATTCAGATG
Y222A	Reverse	CATCTGAATCAGGTAGGCGCCCATCTGCCGTTCTAG
Y223A	Forward	GAACGGCAGATGGGCTACGCCCTGATTCAGATGTATATTC
Y223A	Reverse	GAATATACATCTGAATCAGGGCGTAGCCCATCTGCCGTTC
Y228A	Forward	CTACTACCTGATTCAGATGGCTATTCCCAGCCTGCTCATTG
Y228A	Reverse	CAATGAGCAGGCTGGGAATAGCCATCTGAATCAGGTAGTAG
W239A	Forward	GCTCATTGTCATCCTCTCAGCGATCTCCTTCTGGATCAAC
W239A	Reverse	GTTGATCCAGAAGGAGATCGCTGAGAGGATGACAATGAGC
F242A	Forward	CATCCTCTCATGGATCTCCGCCTGGATCAACATGGATG
F242A	Reverse	CATCCATGTTGATCCAGGCGGAGATCCATGAGAGGATG
W243A	Forward	CTCTCATGGATCTCCTTCGCGATCAACATGGATGCTGC
W243A	Reverse	GCAGCATCCATGTTGATCGCGAAGGAGATCCATGAGAG
W286A	Forward	GTGAAAGCTATTGACATCGCGATGGCTGTTTGCCTGCTC
W286A	Reverse	GAGCAGGCAAACAGCCATCGCGATGTCAATAGCTTTCAC
F293A	Forward	GCTGTTTGCCTGCTCGCCGTGTTCTCGGCCCTG
F293A	Reverse	CAGGGCCGAGAACACGGCGAGCAGGCAAACAGC
F295A	Forward	GCCTGCTCTTCGTGGCCTCGGCCCTGCTGGAATATG
F295A	Reverse	CATATTCCAGCAGGGCCGAGGCCACGAAGAGCAGGC
Y301A	Forward	CTCGGCCCTGCTGGAAGCTGCCGCTGTCAACTTTG
Y301A	Reverse	CAAAGTTGACAGCGGCAGCTTCCAGCAGGGCCGAG
F306A	Forward	GGAATATGCCGCTGTCAACGCTGTGTCTCGGCAACATAAG
F306A	Reverse	CTTATGTTGCCGAGACACAGCGTTGACAGCGGCATATTCC
F395A	Forward	GACAAAATATCCCGCATTGGCGCCCCCATGGCC
F395A	Reverse	GGCCATGGGGGCGCCAATGCGGGATATTTTGTC
F399A	Forward	GGCTTCCCCATGGCCGCCCTCATTTTCAACATGTTC
F399A	Reverse	GAACATGTTGAAAATGAGGGCGGCCATGGGGAAGCC
F402A	Forward	CCCATGGCCTTCCTCATTGCCAACATGTTCTACTGGATC
F402A	Reverse	GATCCAGTAGAACATGTTGGCAATGAGGAAGGCCATGGG
F405A	Forward	GCCTTCCTCATTTTCAACATGGCCTACTGGATCATCTACAAG
F405A	Reverse	CTTGTAGATGATCCAGTAGGCCATGTTGAAAATGAGGAAGGC
Y406A	Forward	CCTCATTTTCAACATGTTCGCCTGGATCATCTACAAGATTGTCCG
Y406A	Reverse	CGGACAATCTTGTAGATGATCCAGGCGAACATGTTGAAAATGAGG
W407A	Forward	CCTCATTTTCAACATGTTCTACGCGATCATCTACAAGATTGTCCG
W407A	Reverse	CGGACAATCTTGTAGATGATCGCGTAGAACATGTTGAAAATGAGG
Y410A	Forward	CATGTTCTACTGGATCATCGCCAAGATTGTCCGTAGAGAGG
Y410A	Reverse	CCTCTCTACGGACAATCTTGGCGATGATCCAGTAGAACATG
