# Supplementary material for: The roles of aromatic residues in the glycine receptor transmembrane domain
Source: BMC Neurosci. 2018 Sep 6;19:53. doi: 10.1186/s12868-018-0454-8 (PMC6127993; doi:10.1186/s12868-018-0454-8)
Supplement: Supplementary file 2 — Additional file 2: Figure S1. Example immunofluorescent images from WT F222A, and F223A GlyR shows all were expressed in HEK293 cells. [file 12868_2018_454_MOESM2_ESM.pdf]

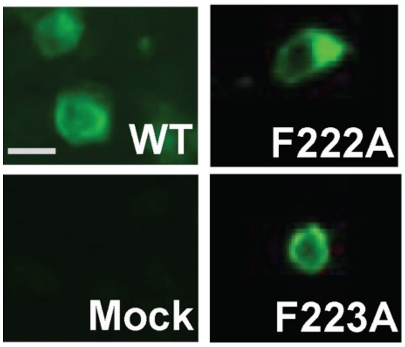

Figure S1: Example immunofluorescent images from WT F222A, and F223A GlyR shows all were expressed in HEK293 cells . Mock = mock transfected cells. Scale bar = 20μm
